# Primary Pancreatic Leiomyosarcoma

**DOI:** 10.5812/iranjradiol.4880

**Published:** 2014-05-15

**Authors:** Ercan Kocakoc, Nuri Havan, Mehmet Bilgin, Musa Atay

**Affiliations:** 1 Department of Radiology, Faculty of Medicine, Bezmialem Vakif University, Istanbul, Turkey

**Keywords:** Pancreatic Neoplasms, Leiomyosarcoma, Multislice Computed Tomography

## Abstract

Primary pancreatic leiomyosarcomas are rare malignant neoplasms with an aggressive course and a large size. A 56-year-old woman presented with an 8-year history of abdominal pain. Multislice computed tomography revealed a large heterogeneous mass with necrotic, calcified and macroscopic fatty areas. The tumor was excised. Histopathological evaluation revealed leiomyosarcoma of the pancreas. If a patient has a large size mass with a cystic-necrotic component, pancreatic leiomyosarcoma should be considered in the differential diagnosis list after excluding other common differential diagnoses.

## 1. Introduction

Leiomyosarcomas are rare malignant tumors of smooth muscle origin (1). They comprise less than 1% of all cancers and 2%-9% of sarcomas (1). They appear mostly in the gastrointestinal tract, retroperitoneum, urinary tract, uterus, and soft tissue (2).

Primary pancreatic leiomyosarcoma is a rare malignant neoplasm. It accounts for 0.1% of all malignant pancreatic cancers (3). Only 45 cases of pancreatic leiomyosarcoma have been reported in the literature. We present the computed tomography findings of primary pancreatic leiomyosarcoma.

## 2. Case Presentation

A 56-year-old woman presented with an 8-year history of abdominal pain especially on the left side. She was otherwise asymptomatic, with no nausea, vomiting, weight loss, diarrhea or jaundice. Laboratory findings were normal except for the increased amylase and erythrocyte sedimentation rate (ESR) level (amylase; 342 U/L, ESR; 71mm/h). The patient had diabetes mellitus (about 5 years, non-insulin dependent type), hypertension and ischemic hearth diseases.

**Figure 1.**
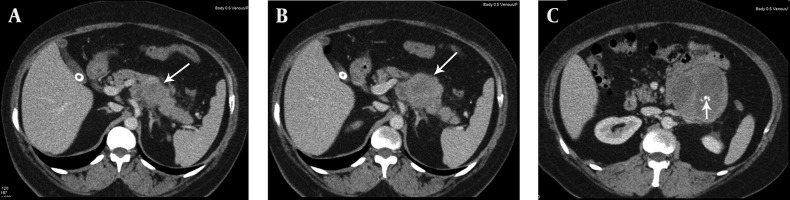
A, B and C) Contiguous portal venous phase axial contrast enhanced CT images show a large heterogeneous mass with central necrotic areas and calcifications (short arrow) arising from the tail of the pancreas (long arrows) extending near the anterior of the left kidney and aorta.

**Figure 2.**
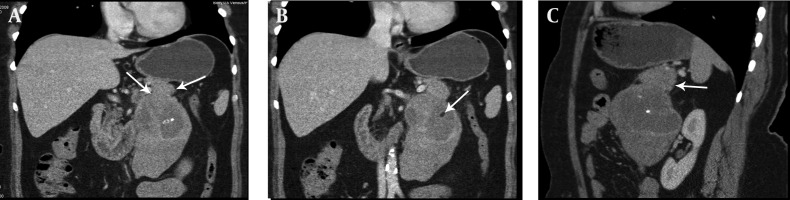
A, B) Coronal and C) Sagittal multiplanar reformatted images show a large exophytic mass containing macroscopic fat (short arrow) extending from the tail of the pancreas (long arrows) to the anterior of the left kidney.

**Figure 3.**
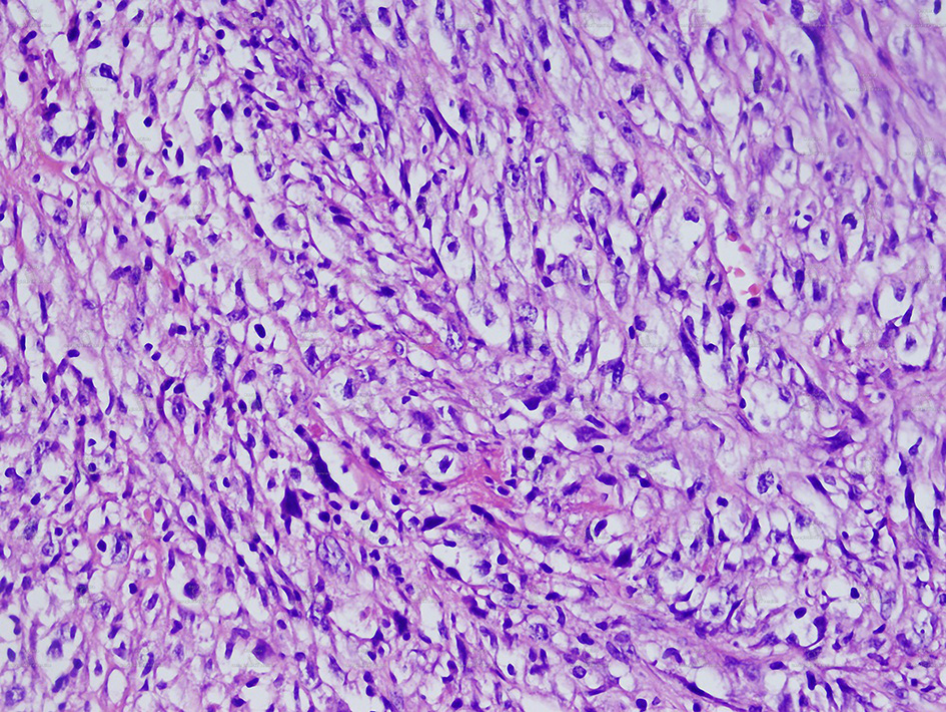
Appearance of atypic cells in leiomyosarcoma. H&E 400×

Chronic pancreatitis was considered clinically and ultrasound (US) examination was requested. US revealed a large heterogeneous hypervascular mass with central necrotic areas near the anterior of the left kidney and aorta. Computed tomography (CT) showed a large heterogeneous mass with necrotic and calcified areas at the pancreatic tail (Figure 1). After contrast administration, marked enhancement was noted. It also contained a small amount of macroscopic fat (Figure 2). Tumor markers (CEA, AFP, CA-125, and CA-15-3) were normal except for a slightly increased CA-19-9 level (27.1 U/mL; normal range 0-18.4 U/mL). Stromal tumor or sarcomatous tumoral lesion was considered. Because neither pathological lymph node nor liver metastasis was detected in the abdominal CT, only postero-anterior chest X-ray was requested for metastasis work-up and it did not show any metastasis. Distal pancreatectomy was performed and an approximate 15×10 cm complex solid mass with cystic components was removed near the left renal vein at the pancreatic tail. Histopathological evaluation revealed stage IIA (T2bN0M0 G1) leiomyosarcoma of the pancreas (Figure 3). No recurrence or residue was detected within one year follow-up.

## 3. Discussion

Primary pancreatic leiomyosarcoma is a rare malignant neoplasm. The mean age is 45.3 (14-83 years). It is not easily differentiated from other pancreatic masses and it mimicks other primary and secondary neoplasms of the pancreas (4). Usual symptoms are epigastric pain, weight loss, jaundice, nausea and vomiting. Incidentally detected cases have also been reported (5-7). Lesions are usually located in the head of the pancreas (involving the entire pancreas in two cases, in the head in 21 cases, in the corpus in nine cases, in the tail in four cases, and in the tail-corpus in one case). It can resemble solid lesions with a cystic component due to necrosis (8, 9). Contrast enhancement is seen in arterial and venous phase CT (5). Due to the large size of the lesion, compression and invasion of neighboring organs (such as the duodenum) can be present (10). Metastasis may be present during first detection.

MRI characteristics of leiomyosarcomas have been described in other organs (11). Most lesions are isointense with skeletal muscle on T1 weighted images, and hyperintense on T2 weighted images. Gadolinium enhancement is usually heterogeneous. Pancreatic leiomyosarcomas have similar imaging characteristics (12). On ultrasound pancreatic leiomyosarcomas appear as a hypoechoic mass to the normal pancreas parenchyma similar to our case (12). Immunohistochemical analyses are of great value in evaluating spindle cell tumors with prominent cellular pleomorphism and/or a focally storiform pattern, supposed to be of smooth muscle origin (13). They reveal positivity for muscle markers (i.e. MSA, a-smooth muscle actin, and desmin) with negative reactions for epithelial (i.e. cytokeratin, EMA, and CEA) and neural (i.e. S100 protein) antigens (13). In our case, the tumor had cellular pleomorphism and a storiform pattern. A-smooth muscle actin marker was positive. 

Usual therapeutic approaches are pancreatoduodenectomy if the lesion is located in the head of the pancreas, and pancreatectomy if the lesion is located in the pancreatic corpus or tail. If the patient has widespread metastasis, surgery is not suitable.

Muhammad et al. demonstrated an enlarged liver containing multiple lesions including the caudate lobe suggestive of metastases and a large enhancing soft tissue mass in the region of the body of the pancreas on CT scan in their patient who was admitted with a one-month history of 16 kilograms weight loss, epigastric pain, anorexia, abdominal fullness and jaundice. After ultrasound-guided liver biopsy, histological and immunohistochemical analysis confirmed leiomyosarcoma of pancreatic origin. The patient was offered palliative chemotherapy because of the metastatic phase. Then the patient died 3 months after the initial diagnosis (9). Nesi et al. reported a large, solid mass replacing the tail of the pancreas, without invasion of the surrounding tissues on CT. After laparotomy, no direct invasion of adjacent structures including retroperitoneal fat was found. Distal pancreatectomy with splenectomy was performed. Then they demonstrated recurrence on follow-up after 9 months. One year after surgery, the patient died (13). Aihara et al. presented a case of a low-density mass located in the body of the pancreas on CT examination. The initial diagnosis was pseudocyst. It was managed conservatively. A repeat CT scan 10 months later showed an increase in the size of the mass. At laparotomy, the tumor arose from the mid-body of the pancreas and there was no invasion into the surrounding organs. Local excision of the tumor was performed (14). In our case, there was a large heterogeneous mass with necrotic calcified areas and a small amount of macroscopic fat at the pancreatic tail. Cases containing macroscopic fat have not been reported before in the literature. Distal pancreatectomy was performed and there was no problem on follow-up in our case. According to the given examples and other cases, primary pancreatic leiomyosarcomas have various appearances, mortalities and treatment options.

Primary pancreatic leiomyosarcomas are rare malignant neoplasms. They show an aggressive course and have a large size. If the patient has a large size mass with a cystic-necrotic component, pancreatic leiomyosarcomas should be considered in the differential diagnosis list after excluding other common differential diagnoses.
